# Control of Capsid Transformations during Reovirus Entry

**DOI:** 10.3390/v13020153

**Published:** 2021-01-21

**Authors:** Stephanie L. Gummersheimer, Anthony J. Snyder, Pranav Danthi

**Affiliations:** Department of Biology, Indiana University, Bloomington, IN 47405, USA; sstupps@iu.edu (S.L.G.); anthsnyd@indiana.edu (A.J.S.)

**Keywords:** reovirus, cell entry, capsid

## Abstract

Mammalian orthoreovirus (reovirus), a dsRNA virus with a multilayered capsid, serves as a model system for studying the entry of similar viruses. The outermost layer of this capsid undergoes processing to generate a metastable intermediate. The metastable particle undergoes further remodeling to generate an entry-capable form that delivers the genome-containing inner capsid, or core, into the cytoplasm. In this review, we highlight capsid proteins and the intricacies of their interactions that control the stability of the capsid and consequently impact capsid structural changes that are prerequisites for entry. We also discuss a novel proviral role of host membranes in promoting capsid conformational transitions. Current knowledge gaps in the field that are ripe for future investigation are also outlined.

## 1. Introduction

Viruses are complex molecular machines that make use of host cell machinery for propagation. Perhaps the most critical part of the infectious cycle is entry into the host cell. Entry is a complicated process that involves not only attachment to viral receptors to initiate uptake but also disassembly of the viral capsid and release of the genome at the appropriate time and cellular location. Viruses have evolved numerous methods of exploiting the host cell machinery to initiate uptake and release of the viral genome to begin replication. Studies of these mechanisms have revealed insights into cellular transport machinery, endosomal characteristics, viral structural components and sorting mechanisms. Here, we will discuss the entry process of a member of the Reoviridae family, mammalian orthoreovirus (reovirus). We will focus on viral and host control of steps that occur following partial disassembly of capsids by host proteases and lead to endosomal escape.

**Figure 1:**
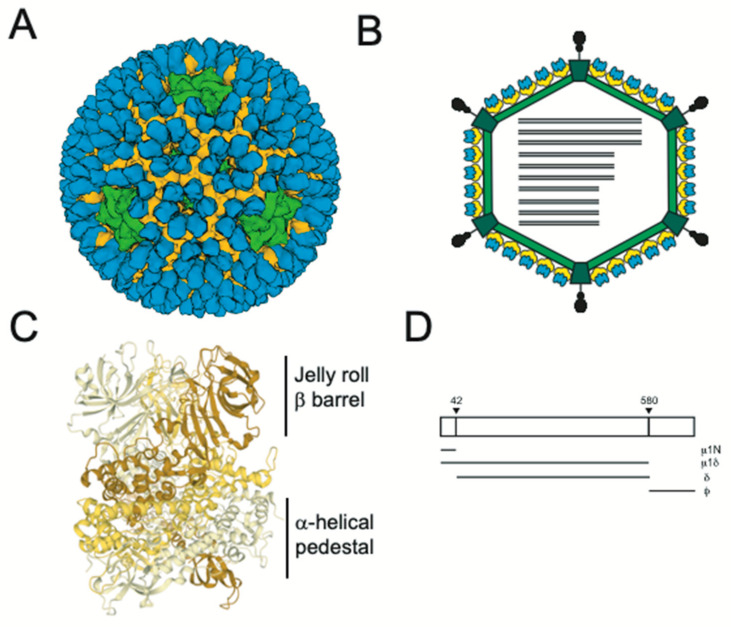
(**A**) Cryoelectron microscopy (cryoEM) structure of reovirus virions rendered from PDB 2CSE (**A**) and its schematic representation (**B**) are shown. Core proteins, μ1, σ3 and σ1 are shown in green, yellow, blue and black, respectively. σ1 is not resolved in the cryoEM structure. (**C**) A trimer of μ1 is shown with each monomer in a shade of yellow. (**D**) Cleavage fragments of the μ1 protein formed during the entry of virus particles into cells are shown.

Reoviruses are non-enveloped viruses with a segmented dsRNA genome. One distinguishing feature of reoviruses is a multilayered capsid. The capsid is composed of two concentric protein shells. The innermost shell, or core, of these viruses contains 10 dsRNA genome segments and remains intact throughout the entirety of replication [1]. The entry process must, therefore, contain an added layer of complexity in which only the outer layer of the particle is lost during the disassembly steps prior to endosomal escape and the entire core is delivered to the host cell cytoplasm. The core of reovirus is composed primarily of λ1 decamers held in place by σ2 clamp proteins [2]. The outer capsid is composed of μ1 trimers, which are capped by σ3 trimers to form 200 µ1-σ3 heterohexamers [3,4]. At each fivefold axis of symmetry, the λ2 turret proteins protrude through both the core and the outer capsid [3,4]. These turrets hold the attachment protein σ1 [3,4] (Figure 1).

**Figure 2:**
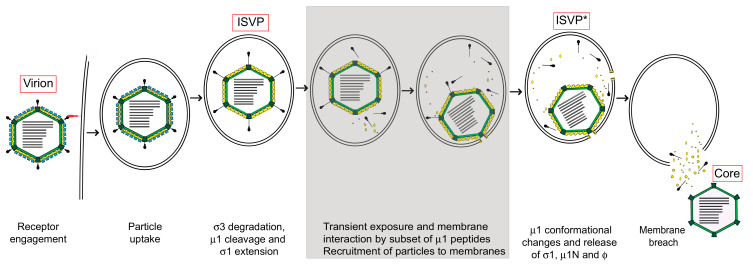
Reovirus entry schematic. Reovirus virions are internalized following engagement to cell surface receptors and disassemble to form infectious subvirion particles (ISVPs) within the endosomal compartment. ISVPs undergo conformational transitions to form ISVP*s and release μ1 fragments that facilitate membrane penetration. We propose (shown in grey boxes) that a small proportion of μ1 fragments are exposed or released from the particles following ISVP formation. These fragments interact with the host membrane and recruit ISVP-like particles to host membranes and promote their conversion to ISVP*s. Red boxes indicate key virus entry intermediates.

The initial entry events of reovirus, including attachment and internalization pathways, have been extensively reviewed elsewhere and will not be covered in great detail in this review [5,6]. In brief, attachment to host cells is mediated by the viral σ1 protein. Interactions between σ1 and glycans have been identified in multiple serotypes of reovirus [7]. Each reovirus serotype uses junction adhesion molecule A (JAM-A) as a host cell receptor [8,9]. In some cell types, the Nogo-66 receptor NgR1 could act as a receptor by interacting with an as-yet-unknown viral component [10]. After attachment, particles are endocytosed in a clathrin- or caveolin- dependent manner and sorted into early and late endosomes [11] (Figure 2). This process may be dependent on β1 integrins [12,13]. In some cell types, uptake occurs via macropinocytosis [14]. Disassembly occurs while the particle is in the endosome and is mediated by endosomal acid-dependent cysteine proteases to generate the infectious subvirion particle, or ISVP [15]. ISVPs are characterized by the loss of σ3, conformational changes in σ1 and proteolytic cleavage of μ1 into particle-associated ϕ and μ1δ fragments [16]. In some tissues, particularly in the enteric tract and the lungs, disassembly can also occur extracellularly by the action of proteases present in the lumen [17,18,19]. Extracellular disassembly produces particles that are indistinguishable from ISVPs that are produced in cells. The ISVP is a metastable entry intermediate poised to undergo additional changes that are necessary for delivering the core particle across the host membrane (Figure 2).

In this review, we will discuss steps that follow the generation of ISVPs. These include the extensive rearrangements of the outer capsid proteins that allow for the release of specific peptides to facilitate cell penetration. We will discuss the important roles attributed to the µ1 protein during these early events that are key for entry and endosomal escape. We will also discuss a role for host lipid components in these processes. Finally, we will summarize new data that suggest unexpected roles for additional viral capsid proteins in controlling the metastability of the particle that impact the kinetics and efficiency of cell entry.

## 2. Structural Changes Required for Membrane Penetration Are Controlled by the μ1 Outer Capsid Protein

The μ1-σ3 heterohexamers that make up the outer capsid are organized into an incomplete T = 13 lattice (Figure 1). The σ3 protector protein caps the μ1 cell penetration protein, presumably to prevent premature disassembly. Each μ1 monomer is characterized by a jelly roll β-barrel domain that connects to a core-adjacent α-helical pedestal; three monomers intertwine to form a trimer (Figure 1) [3,20]. There also exist interactions between neighboring trimers and between trimers and core proteins, particularly σ2 and λ2. As described above, σ3 is degraded in the endosome to generate a metastable intermediate called the infectious subvirion particle (ISVP), in which μ1 becomes solvent-exposed (Figure 2) [4]. At their current resolutions, the conformations of µ1 in virions and in ISVPs are indistinguishable [4]. ISVPs then undergo an irreversible transition to deposit the genome-containing core into the host cytoplasm. μ1 is cleaved to generate the μ1N (residues 2 to 42), δ (residues 43 to 580) and ϕ (residues 581 to 708) fragments. δ subsequently adopts a hydrophobic and protease-sensitive arrangement, whereas μ1N and ϕ are released and form the pore complex [21,22,23,24]. The release of μ1N and ϕ requires the separation and unwinding of μ1 trimers [25], thus generating a particle with an altered conformation, called ISVP* [21] (Figure 2). Although these biochemical changes are well characterized, no structural information is available for ISVP*. ISVP-to-ISVP* conversion can be triggered in vitro using non-specific factors such as heat, red blood cells or large monovalent cations [21,26]. When generated outside the context of a cell, ISVP*s are non-infectious—likely due to loss of the attachment protein and the membrane penetration machinery [26]. σ3-associated µ1 can also adopt an altered conformation reminiscent of ISVP*s, concurrent with inactivation [27]. Nonetheless, this transition only occurs following exposure to high temperature or ethanol [27]. Additional factors that regulate disassembly include virus strain-specific effects (discussed below) [28], protease activity during virion-to-ISVP conversion [29], the pH of the endosomal compartment [30] and the presence and concentration of lipid-associated μ1N, which promotes uncoating through a positive feedback loop (discussed below) [31,32]. It remains unknown if a specific cellular factor triggers ISVP-to-ISVP* conversion during infection.

Comparing ISVPs of prototype reovirus strains type 1 Lang (T1L) and type 3 Dearing (T3D), differences in thermostability and hemolytic capacity (which correlate with the efficiency of ISVP-to-ISVP* conversion) map to the M2 gene segment (which encodes for μ1) [21,28]. Within μ1, the C-terminal portion of δ, which corresponds to the jelly roll β-barrel domain, is key to controlling these activities (Figure 1) [28]. Numerous studies examined the impact of single- or multi-point mutations on heat resistance. Stabilizing changes in δ include D371A, G383E, K459E, D541N (within T1L μ1), A276V, A305V, A319E, V403A, K407R and I442V (within T3D μ1) [29,33,34,35,36]. Interestingly, K594D, T612M, A615T and P697Q, which are within the ϕ portion of μ1, also enhance capsid integrity [33,37,38]. In contrast, destabilizing changes in δ include S134L, A184V, P277T (within T1L μ1),E89A/Q, and Δ341/342 (within T3D μ1) [35,36,39]. Many of the above mutations, particularly those that fall within δ, lie within or adjacent to μ1-mediated intratrimer, intertrimer or trimer–core interactions. Thus, they likely influence ISVP stability by impacting the propensity for the separation and unwinding of μ1 trimers. Residues outside of this region may regulate ISVP stability by affecting the release of μ1N, which promotes ISVP* formation (described below), or via allosteric effects. As such, there exists a balance between environmental stability and conformational flexibility. µ1 must maintain structural integrity; however, the lattice must also undergo efficient uncoating during cell entry. In some cases (e.g., T1L K459E, T3D K407R and T3D Δ341/342), altered stability is correlated with impaired replication and/or spread [33,36]. Certain hyperstable variants (e.g., T3D I442V and T3D K594D) have also been tested in vivo. These viruses replicate less efficiently and were attenuated in a newborn mouse model [34,37].

## 3. Target Membranes Control Structural Transitions in the Virus Particle

Viruses adopt metastable conformations prior to or during cell entry. To perforate the host membrane barrier, one or more cellular cues reduce the energy barrier for viral proteins to refold into a membrane penetration-active form. Entry pathways that require interactions with host proteins or low pH are well characterized [40,41]; however, knowledge of alternative triggers is limited. For reovirus, synthetic membranes composed of phosphatidylcholine and phosphatidylethanolamine induce ISVP-to-ISVP* conversion in vitro [42]. Moreover, this process occurs most efficiently in the presence of lipid-associated μ1N, which recruits ISVP*s to membrane pores [31]. Thus, we propose a model in which host membranes actively participate in the entry pathway of reovirus. Partially converted ISVP*s are formed by “breathing” motions within the outer capsid, resulting in the transient exposure of buried regions of µ1 and a small proportion of µ1N. Transiently exposed µ1 then interacts with lipid-associated μ1N, triggering further changes by a positive feedback loop to form ISVP*s. Subsequently, pores are formed to facilitate core delivery (Figure 2) [23,24,43]. This model may preclude the need for additional cellular factors to mediate ISVP-to-ISVP* conversion. Such dynamic movements have been demonstrated with or predicted for other virus systems [44,45,46,47]. Though host lipid composition has been implicated in the uptake of adenovirus or in the formation of pores by bluetongue virus (BTV) [48,49], to our knowledge, the impact of lipids on controlling structural transitions of capsids is unique to reovirus.

Among related dsRNA viruses with multilayered capsids that are well studied, the triggers for conformational changes in the outer capsid that precede cell membrane penetration appear distinct from those used by reovirus. For bluetongue virus (BTV), low pH triggers conformational changes in the virus in two stages as the virus transits through the increasingly acidified compartments of the endosomal pathway [50,51]. In early endosomes, the receptor-bound BTV VP2 protein senses low pH via a histidine residue and is detached from the particle exposing the VP5 outer capsid protein. The VP5 protein can also sense the lower pH of the late endosome via a cluster of histidine residues and unfurl, such that newly exposed regions interact with membranes and form pores that facilitate membrane penetration. For rotavirus, there also appear to be two stages of conformational changes that promote membrane penetration. The first step for some rotavirus strains is low-pH-dependent and results in a change in the conformation of VP5* (a proteolytically generated cleavage fragment of the VP4 protein) [52]. For other strains which can enter without the need for endosomal acidification, the trigger is unknown. Nonetheless, under these conditions, VP5* is able to interact with the membrane. The second change requires destabilization and removal of the VP7 layer, which occurs as a consequence of a decrease in the concentration of calcium ions when the virus is within the endosomal compartment [53,54]. Removal of VP7 allows VP5* to assume a folded-back conformation that results in the disruption of the host membrane [55,56].

It should also be noted that for fusogenic members of the *Reoviridae* family, outer capsid-dependent entry may be dispensable following initiation of infection of the first cell in a population. These viruses encode a fusion-associated small transmembrane (FAST) protein [57]. FAST proteins are small, single-pass membrane components that are not incorporated into virions. FAST proteins mediate cell–cell fusion and syncytium formation [58], thus contributing to localized spread of virus infection, perhaps without the need to form multilayered capsids or release particles from infected cells.

## 4. Role for Other Reovirus Capsid Proteins in Controlling Conformational Changes that Precede Cell Penetration

As described above, the roles for µ1 and its cleavage fragments in controlling entry events are well characterized. Recent data gathered using reassortant viruses have shown that other proteins, both in the core and the outer capsid, also contribute to ISVP stability and therefore impact the efficiency of infection initiation.

### 4.1. Role for σ1 and λ2

There are a maximum of 36 copies of the attachment protein σ1, which are present as homotrimers, on each reovirus virion. Turrets made up of λ2 pentamers at each fivefold axis of symmetry hold the σ1 trimers [4]. σ1 mediates binding to receptors on the cell surface. During ISVP formation, σ1 undergoes conformational changes and assumes an extended state [4,59]. σ1 is ejected from the particle upon conversion of the particle to an ISVP*, likely due to changes in the structure of the λ2 pentamers [21]. The eventual fate of σ1 following its release is unknown. Work using reassortant viruses engineered in a laboratory indicates that σ1 and λ2, and their interactions with other outer capsid proteins, can play additional unexpected roles during entry. A reassortant virus (T3D^F^/T3D^C^ S1) which contains nine gene segments from the laboratory strain T3D^F^ and the S1 gene segment (encoding σ1) from the laboratory variant T3D^C^ demonstrated lower stability and more efficient ISVP-to-ISVP* transition in comparison to T3D^F^ [60]. Thus, properties of σ1 can affect the stability of ISVPs. The T3D^F^ and T3D^C^ σ1 proteins differ by only two amino acids. One of the polymorphisms within σ1 is within a region of σ1 that interacts with λ2, suggesting that a mismatch between σ1 and λ2 impacts ISVP stability. A double reassortant (T3D^F^/T3D^C^ S1 L2) displayed intermediate ISVP-to-ISVP* conversion efficiency between that of T3D^F^ and T3D^F^/T3D^C^ S1, supporting this idea. The σ1 protein makes contacts with λ2. Thus, changes in the position of λ2 residues due to the presence of polymorphic variations between strains may alter their interaction with µ1 and thereby affect the ease with which µ1 and other parts of the capsid undergo rearrangement during ISVP* formation. Although σ1 does not contact µ1, changes in σ1–λ2 interaction may, in turn, affect λ2 in such a way that its association with µ1 is affected. Thus, it appears that the proper assembly of the outer capsid impacts its stability and the propensity to generate ISVP*s.

### 4.2. Role for σ2 and λ1

The core is made up of 120 copies of λ1, which are held in place by 150 copies of the σ2 clamp protein (encoded by the L3 and S2 gene segments, respectively) [38]. Core particles remain intact throughout replication and are not degraded during entry. Therefore, it was not expected that proteins that comprise the core would play a significant role in ISVP-to-ISVP* conversion. However, recent data demonstrated that a reassortant virus containing eight gene segments from T3D^F^ and the S2 and L3 gene segments from the strain T1L showed diminished stability and enhanced ISVP-to-ISVP* conversion compared to a WT T3D^F^ virus [38]. Thus, assembly of the core proteins also impacts ISVP stability, thereby affecting the efficiency with which ISVP*s are formed. This work further connects assembly of the virus to ISVP* formation and suggests that the metastability of the reovirus outer capsid is affected by core proteins that are thought to remain static during cell entry.

The reovirus capsid is comprised of eight proteins [4]. Each of these proteins performs a critical function in the reovirus replication cycle (such as attachment by σ1 or mRNA capping by λ2). Until recently, studies on reassortant or mutant viruses were limited to evaluating the impact of a protein on its known function. For example, a σ1 mutant or reassortant is characterized for its impact on attachment. It is important to recognize that each of these proteins is part of a multi-protein complex (the two-layered capsid) which is assembled with intricate geometry. Thus, a change in one of these proteins can impact how the capsid is held together. Because many of the reovirus capsid proteins only function in the context of a capsid, changes in assembly fidelity can impact the functions of the capsid. Here, we provided examples that suggest a need for examining reassortant or mutant viruses with a broader lens to ensure that all phenotypes are identified and accounted for before drawing conclusions.

Reoviruses are one among many segmented viruses capable of undergoing reassortment. Particles of segmented viruses such as those within the orthomyxovirus or arenavirus families form enveloped particles with pleomorphic structures that contain a variable number of protein subunits [61,62]. In contrast, segmented viruses that are non-enveloped form icosahedral structures that are comprised of a precise arrangement of a fixed number of capsid protein subunits. When viruses with such capsid architecture reassort, alleles of structural proteins that are independently evolved are placed together. When propagated as a clone, mismatched capsid subunits within a reassortant are forced to interact with each other and may produce unexpected phenotypes that highlight previously unrecognized roles for capsid protein in other aspects of viral biology, similar to those that we have noted for reovirus. Yet, few, if any, data exist for even the more extensively studied segmented non-enveloped viruses such as rotavirus and BTV. Therefore, fascinating opportunities exist for additional research into the possible unexpected effects of reassortment across multiple virus fields.

## 5. Conclusions

In this review, we discussed the roles of various capsid proteins during reovirus entry. The µ1 protein and its cleavage fragments play significant roles during ISVP* formation and cell membrane penetration. Nonetheless, our knowledge of these functions is incomplete. µ1 undergoes major conformational changes during cell entry which allow for the release of the µ1N and ϕ fragments. µ1 residues that mediate interactions within and between µ1 trimers control ISVP-to-ISVP* conversion. µ1 residues that do not fall into either of these criteria have also been implicated but how these residues impact ISVP* formation is unknown. While biochemical changes in µ1 during entry have been interpreted to indicate structural changes, the precise arrangement of µ1 in its altered state remains undescribed. Changes to other parts of particles that occur concomitantly with ISVP* formation are also unidentified. The µ1N fragment promotes the conversion of ISVPs to ISVP*s. Though the ISVP*-promoting activity of µ1N is most efficient in the presence of host membranes, the precise role of host membranes in this process remains undefined. Enhancement of ISVP* formation by µ1N occurs via recruitment of ISVP-like particles to the membrane. Yet, neither the residues in µ1N that are important for particle recruitment nor the portion of ISVPs that interact with µ1N have been identified. The cleavage of the ϕ fragment is required for efficient membrane penetration. Nonetheless, how µ1N and ϕ form pores that deliver core particles across the membrane is unclear.

We also discussed the roles played by σ1, σ2, λ1 and λ2 capsid proteins in controlling particle metastability and, consequently, the formation of ISVP*s. Among these, σ2 makes direct interactions with µ1 on the outer capsid. Thus, it is likely that the strength of the interaction between σ2 in the core and µ1 on the outer capsid regulates the propensity with which µ1 can undergo conformational changes. λ1 may indirectly impact capsid stability as a consequence of its interaction with σ2. λ2 also makes interactions with µ1 and, thus, may similarly impact ISVP* conversion. Additionally, based on the evidence that ISVP-to-ISVP* change is accompanied by the release of σ1 and the activation of the transcriptional machinery within the core, it is assumed that λ2 also undergoes a conformational change that resembles the more open structure present in cores. Thus, another way in which λ2 may impact ISVP* formation may be as a consequence of its own capacity for structural changes. The impact of σ1 on ISVP-to-ISVP* conversion may be due to its interaction with λ2. Nonetheless, this work suggests that even subtle changes to particle structure affect these events. Such studies support the idea that the assembly properties of the virion affect capsid metastability, thereby regulating the efficiency of cell entry. The critical protein–protein interactions within the reovirus capsid that are key to maintaining this balance remain to be identified.

## Data Availability

No new data were created or analyzed in this study. Data sharing is not applicable to this article.
